# Circulating tumour cells and tumour biomarkers in functional midgut neuroendocrine tumours

**DOI:** 10.1111/jne.13096

**Published:** 2022-02-07

**Authors:** Tim Meyer, Martyn Caplin, Mohid S. Khan, Christos Toumpanakis, Shishir Shetty, John K. Ramage, Aude Houchard, Kate Higgs, Tahir Shah

**Affiliations:** ^1^ University College London London UK; ^2^ Royal Free Hospital London UK; ^3^ 97609 University Hospital of Wales Cardiff UK; ^4^ 156807 Queen Elizabeth Hospital Birmingham Birmingham UK; ^5^ Kings College Hospital London and Hampshire Hospitals London UK; ^6^ Ipsen Pharma Boulogne‐Billancourt France; ^7^ Ipsen Slough UK

**Keywords:** circulating tumour cells, lanreotide autogel, neuroendocrine tumours, plasma 5‐hydroxyindoleacetic acid, urinary 5‐hydroxyindoleacetic acid

## Abstract

CALM‐NET was a phase IV exploratory study in the UK that aimed to evaluate if the presence of circulating tumour cells (CTCs) at baseline predicted symptomatic response in patients with midgut neuroendocrine tumours (NETs) treated with lanreotide autogel (LAN). Adults with functional, well/moderately differentiated (Ki‐67 <20%) midgut NETs received LAN 120 mg/28 days for 1 year. CTCs were present in blood if enumeration was >0. Primary endpoint was the clinical value of baseline CTCs to predict symptomatic response (decrease in diarrhoea or flushing of ≥50% frequency, or ≥1 severity level). Other endpoints included progression‐free survival (PFS) and correlations between plasma and urinary biomarkers (including 5‐hydroxyindoleacetic acid [5‐HIAA]). Fifty patients were enrolled; 40 completed the study. Baseline CTCs were present in 22 (45.8%) patients (missing baseline CTC status *n* = 2). Overall, 87.5% (95% confidence interval [CI]: 73.9; 94.5) of patients had a symptomatic response; a 5.9‐fold higher odds of symptomatic response in patients without CTC versus patients with CTC at baseline was observed, although this was not statistically significant (odds ratio: 0.17 [95% CI: 0.02; 1.65], *p* = .126). One‐year PFS rate was 66.4% (95% CI: 48.8; 79.2). Biomarker concentrations did not correlate to baseline CTC status. However, there was a strong correlation between plasma and urinary 5‐HIAA (Spearman correlation coefficients ≥0.87 [*p* < .001], all time points). In conclusion, patients without CTC at baseline may be more likely to achieve a symptomatic response following LAN treatment than patients with CTC. Plasma 5‐HIAA correlated with urinary 5‐HIAA during LAN treatment. ClinicalTrials.gov identifier: NCT02075606.

## Graphical abstract

CALM‐NET was a phase IV exploratory study designed to evaluate if the presence of circulating tumour cells (CTCs) could be used to predict symptomatic response in patients with midgut neuroendocrine tumours (NETs) treated with lanreotide autogel (LAN). The results of the primary endpoint, clinical value of baseline CTCs to predict symptomatic response, showed that patients who were CTC‐negative achieved a response rate of 95.5%, compared with 77.8% for patients who were CTC‐positive (odds ratio: 0.17, 95% CI: 0.02; 1.65). The symptomatic response did not differ between patients with baseline CTCs and those without; however, the study was limited as a result of its small sample size. Clinical symptomatic response rates (flushing and diarrhoea) in LAN‐treated patients according to CTC status. ^a^ Two patients had missing CTC data and eight patients had missing data for clinical symptomatic response (four who were CTC+ and four who were CTC−). Error bars depict the 95% CI. CI, confidence interval; CTC, circulating tumour cell; LAN, lanreotide autogel.

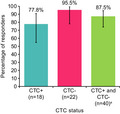

## 1 INTRODUCTION

Neuroendocrine tumours (NETs) are a rare group of tumours originating from neuroendocrine cells occurring most commonly in the gastrointestinal tract (11%–41%),^1^ pancreas, pulmonary bronchi and thyroid gland. In England between 2013 and 2014, the estimated annual incidence of neuroendocrine neoplasms (neuroendocrine carcinoma and NETs) was approximately 8 per 100,000.^2^ NETs are characterized by the overexpression of somatostatin receptors (SSTRs), of which SSTR2 and SSTR5 are highly expressed in midgut NETs.^3^ The management of NETs can be challenging due to the typically late diagnosis and lack of biomarkers to guide treatment, though advances have been made in expanding treatment options.^4^, ^5^ Tests for the classic single‐analyte biomarkers, such as urinary 5‐hydroxyindoleacetic acid (5‐HIAA), chromogranin A (CgA) and neurokinin A (NKA), have inadequate specificities and sensitivities for diagnosis and a lack of predictive value, necessitating the development of novel predictive biomarkers.^4^, ^6^

The presence of circulating tumour cells (CTCs) has been shown to predict survival outcomes in a number of solid tumour malignancies following treatment, including metastatic breast, colorectal, prostate, and lung cancer.^7^, ^8^, ^9^, ^10^, ^11^, ^12^ CTCs have been shown to be detectable in approximately 50% of patients with metastatic NETs, and in these patients, progression‐free survival (PFS) and overall survival were shorter than in those without CTCs; CTC number was also found to correlate with tumour burden, grade and CgA.^13^, ^14^ Reductions in CTC levels post‐treatment have been associated with slower disease progression and improved survival in patients with NETs.^15^ There is increasing interest in the use of CTCs to inform treatment decisions and monitor tumours during treatment, as analysis is relatively noninvasive compared to biopsy, and results can be returned quickly.^7^

Lanreotide autogel (LAN) is a somatostatin analogue (SSA) that has antiproliferative effects through direct activation of SSTRs on tumour cells. LAN has been shown to improve symptoms and PFS in patients with functional NETs when administered at doses of up to 120 mg every 28 days.^16^, ^17^, ^18^, ^19^ LAN prolongs PFS in those with nonfunctional, locally advanced or metastatic enteropancreatic NETs.^20^ The aim of the CALM‐NET exploratory study was to determine whether pretreatment CTC status can help predict the clinical symptomatic response to LAN in patients with functional midgut NETs; CTCs were also enumerated during the study.

## 2 METHODS

**FIGURE 1:**
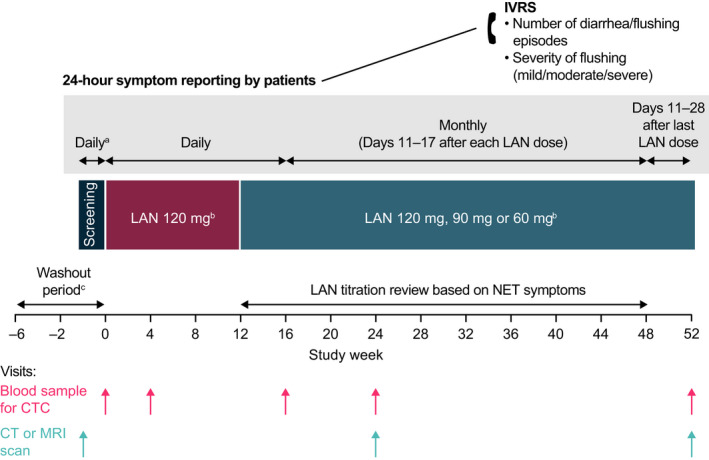
CALM‐NET study design. ^a^For patients not undergoing a washout period. ^b^LAN injection administered once every 28 days. ^c^A washout period is required for patients previously receiving either s.c. octreotide (at least 2 weeks) or one injection of a long‐acting SSA (at least 6 weeks) prior to study entry. For patients undergoing washout, information on symptoms at diagnosis and during prior SSA treatment was obtained from the medical notes of patients. CT, computed tomography; CTC, circulating tumour cell; IVRS, interactive voice response system; LAN, lanreotide autogel/depot; MRI, magnetic resonance imaging; NET, neuroendocrine tumour; s.c., subcutaneous; SSA, somatostatin analogue

CALM‐NET was a 52‐week, phase IV, prospective, open‐label, exploratory study conducted at 11 centres in the UK (EudraCT identifier 2013‐002194‐22; ClinicalTrials.gov identifier: NCT02075606) (Figure 1). The study was approved by an Independent Ethics Committee (London – South East and London – City and East Research Ethics Committee) and conducted under the provisions of the Declaration of Helsinki, in accordance with the International Council for Harmonisation Consolidated Guideline on Good Clinical Practice, and all patients provided written informed consent.

### 2.1 Study population

Patients were eligible for inclusion if they were aged ≥18 years, had a documented diagnosis of functional well‐ or moderately differentiated (Ki‐67 <20%) midgut NETs and had ongoing diarrhoea and/or flushing at enrolment. Eligible patients also had positive SSTR imaging and were clinically suitable for SSA treatment. Patients being treated with a short‐acting SSA and those who had received a single injection of a long‐acting SSA could be enrolled into the study, provided there was an adequate washout period (≥2 weeks for short‐acting subcutaneous octreotide and ≥6 weeks for long‐acting SSAs). Those treated with interferon, chemotherapy, chemoembolization or radionuclide therapy in the previous 3 months were excluded.

### 2.2 Study treatment and concomitant medication

Patients were treated with LAN every 28 days for up to 1 year (48 weeks, followed by 4 week follow‐up period). The dose for the first three injections was 120 mg per injection; thereafter, the dose could be reduced to 90 mg or 60 mg per injection, depending on the clinician's decision, LAN tolerability and the review of the patient's symptoms, as per the summary of product characteristics.^21^ Concomitant medications for acute symptomatic episodes (e.g., loperamide, nifuroxazide or octreotide infusion) were permitted during the study for ethical reasons. Information on their use was recorded in patients' case report forms and taken into account in the primary endpoint analysis.

### 2.3 Study assessments

Patients self‐reported their symptoms by telephone via an interactive voice response system (Figure 1), by answering pre‐set questions to record symptom frequency (“how many times have you had diarrhoea in the last 24 h?”; “how many times have you had flushing in the last 24 h?”) and severity of flushing (“overall, how would you rate your flushing events in the last 24 h? Please give an average rating if more than one flushing event occurred”). Severity of flushing was recorded using a three‐point system: mild, moderate or severe. Patients reported 24‐h symptom frequency and flushing severity on a daily basis for the first 16 weeks of the study. Patients then reported these parameters on Days 11 to 17 of each subsequent injection interval until week 48. After the final study‐drug injection at week 48, patients provided these parameters on a daily basis on Days 11 to 28 post‐injection.

Blood samples were collected from patients for CTC enumeration and the measurement of plasma CgA, 5‐HIAA and NKA concentrations, and 24‐h urine samples were collected for assessment of urinary 5‐HIAA concentration, at baseline and weeks 4, 16, 24, and end of study/early withdrawal (Figure 1). Enumeration of CTCs was conducted using the CellSearch system (Veridex) as previously described.^22^ Patients underwent computed tomography (CT) or magnetic resonance imaging (MRI) scans at screening, week 24, and end of study/early withdrawal (Figure 1). Investigators reviewed the scan images locally to determine disease progression according to response evaluation criteria in solid tumours 1.1 criteria. NETs were staged using the European Neuroendocrine Tumour Society (ENETS) classification criteria, from stage I to IV.

### 2.4 Study endpoints

The primary endpoint was the clinical value of enumeration of CTCs at baseline to predict symptomatic response to LAN. A symptomatic response was assessed as a qualitative variable (yes/no) defined as a reduction in the number of self‐reported daily diarrhoea and/or flushing episodes by ≥50% and/or a decrease in the most commonly reported category of severity (mild, moderate or severe) of flushing by at least one level, between baseline (defined as the 7 days preceding the first study treatment injection) and last period (defined as Days 11 to 17 after the last injection [or after theoretical injection if the patient performed the last visit without injection]).

An additional exploratory primary efficacy endpoint was the ability of CTCs to predict PFS in patients receiving LAN.

Secondary endpoints included CTC enumeration during the study; the effect of LAN on the symptoms of diarrhoea and flushing; and PFS at 1 year of treatment with LAN. The change from baseline of health‐related quality of life (HRQoL) was also assessed using the European Organisation for the Treatment of Cancer (EORTC) Quality‐of‐Life Questionnaire Core 30 (QLQ‐C30) and the EORTC Quality‐of‐Life Questionnaire for Gastrointestinal Neuroendocrine Tumours (QLQ‐G.I.NET21).

Exploratory secondary efficacy endpoints included the correlation between biomarker concentrations (plasma CgA, urinary and plasma 5‐HIAA and NKA) and CTC presence in the overall patient population.

Post hoc analyses were conducted to describe the effect of LAN on plasma CgA, urinary and plasma 5‐HIAA and NKA levels (change from baseline based on the 95% confidence intervals [CIs]) in the overall patient population and in patients with elevated biomarker levels at baseline (>2 × upper limit of normal [ULN]); correlations between plasma 5‐HIAA and urinary 5‐HIAA, CgA and NKA; the ENETS disease stage according to CTC status at baseline; symptomatic response according to ENETS disease stage at baseline; and CTC enumeration over time according to symptomatic response and radiological response.

### 2.5 Statistical analyses

A sample size of 50 patients was chosen for this exploratory study based on practical considerations relating to the number of patients who could be enrolled within the timeframe. It was estimated that this sample size would give 80% statistical power to detect a difference in clinical response rates of approximately 40%; for example, 55% in patients with no measurable CTCs (CTC−) versus 15% in patients with measurable CTCs (CTC+).

For the primary endpoint, clinical symptomatic response was described according to CTC presence (CTC+) at baseline and overall using descriptive qualitative statistics including mean 95% confidence intervals (CIs), and was analysed using univariate logistic regression. The relationship between CTC presence and clinical symptomatic response was also assessed adjusting for the presence of other potentially influential patient characteristics (i.e., age, sex, body mass index, time since NET diagnosis, location of primary tumour, ENETS disease staging, prior medication, and type of concomitant medications potentially impacting symptoms) using univariate logistic regression to identify parameters significant at the 20% level. The potential impact of concomitant medication on the primary endpoint was evaluated according to whether the medication was likely to have a “full” or “partial” effect on symptoms. Medications defined as “antipropulsive”, “somatostatin and analogues” or “other intestinal anti‐infectives” were considered to have a potential effect on symptoms. If such medications were started before the start of the last period of symptom collection (Days 11 to 17 after the last injection) and ongoing during the last period of symptom collection, they were considered to have a potential ‘full effect’ on symptoms. Medications that were not started before the start of the last period of symptom collection and were not ongoing during the last period of symptom collection were considered to potentially have a ‘partial effect’ on symptoms.

Progression‐free survival was analysed using the Kaplan–Meier method; hazard ratios and corresponding 95% CIs were calculated using a Cox proportional hazard model. All other data were summarized using descriptive qualitative statistics. Correlations between plasma 5‐HIAA and other biomarkers were assessed using the Spearman correlation coefficient, and correlation between biomarkers and CTC presence at each time point was assessed using the Wilcoxon test.

## 3 RESULTS

### 3.1 Baseline characteristics

Overall, 50 patients were enrolled and 40 (80.0%) completed the study. Reasons for withdrawal were adverse events (AEs; *n* = 5; ongoing severe diarrhoea; liver failure; lump at injection site; chest pain, and hypoglycaemia), withdrawal of consent (*n* = 1) and other reasons (*n* = 4; investigator's decision; symptom management; increasing symptoms with LAN administered every 3 weeks; death). Baseline demography and clinical characteristics are summarized in Table 1; among patients with data available (*n* = 48), 22 (45.8%) were CTC+ and 26 (54.2%) were CTC− at baseline. At baseline, 36 patients had available data on ENETS disease stage (Table 1); of those patients who were CTC+ at baseline, 78.6% had ENETS stage IV disease and 21.4% had ENETs stage IIa to IIIB disease (post hoc). CT or MRI scans were performed at baseline as part of the screening process; however, ENETS disease staging data were not available for the remaining 14 patients.

**TABLE 1:** Baseline demography and clinical characteristics

Characteristic	Patients (*N* = 50)
Mean (SD) age, years	63.4 (8.6)
Male, *n* (%)	27 (54.0)
Race, *n* (%)	
Caucasian/White	45 (90.0)
Black/African‐American	3 (6.0)
Asian	2 (4.0)
Median (95% CI) time since NET diagnosis, months^a^	4.50 (2.80; 6.58)
Primary tumour, *n* (%)	
Ileum	24 (48.0)
Caecum	5 (10.0)
Jejunum	4 (8.0)
Small bowel	3 (6.0)
Duodenum (second part)^b^	1 (2.0)
Ascending/right colon	1 (2.0)
Appendix	1 (2.0)
Other	5 (10.0)
Unknown^c^	6 (12.0)
Positive somatostatin‐receptor imaging, *n* (%)	50 (100)
ENETS disease stage, *n* (%)	
Missing	14
IIa	1 (2.8)
IIb	2 (5.6)
IIIa	1 (2.8)
IIIb	4 (11.1)
IV	28 (77.8)
Proliferation index, *n* (%)	
≤2%	31 (62.0)
>2 to ≤5%	11 (22.0)
>5 to <10%	4 (8.0)
≥10%	4 (8.0)
Previous SSA treatment	
Octreotide^d^	11 (22.0)
LAN	7 (14.0)
Median (95% CI) number of diarrhoea episodes per day^e^	1.3 (0.6; 2.7)
Median (95% CI) number of flushing episodes per day^e^	2.4 (1.5; 3.1)
Mode severity of flushing, *n* (%)	
None	7 (14.0)
Mild	19 (38.0)
Moderate	23 (46.0)
Severe	1 (2.0)
CTC status, *n* (%)	
Missing	2
Positive	22 (45.8)
Negative	26 (54.2)
CTC+ by ENETS disease stage, *n* (%)	
Missing	8
Stage IV	11 (78.6)
Other stages^f^	3 (21.4)

Abbreviations: 5‐HIAA, 5‐hydroxyindoleacetic acid; CI, confidence interval; CTC, circulating tumour cell; ENETS, European Neuroendocrine Tumour Society; LAN, lanreotide autogel; NET, neuroendocrine tumour; SD, standard deviation; SSA somatostatin analogue.

^a^ At study entry.

^b^ Although the tumour was not of midgut origin, it was 5‐HIAA secreting, and the patient was included in the study and is part of the analysis.

^c^ Location of the primary tumour in the midgut was not documented; however, all patients had a documented diagnosis of midgut NET.

^d^ Eight patients had received previous treatment with subcutaneous octreotide, two patients with octreotide LAR, and one patient had been treated previously with both. No other prior treatments for NETs beyond SSA, were recorded in eligible patients.

^e^ Average number of episodes in 7 days before first study treatment.

^f^ CTC status missing for one patient.

### 3.2 Primary endpoint

**FIGURE 2:**
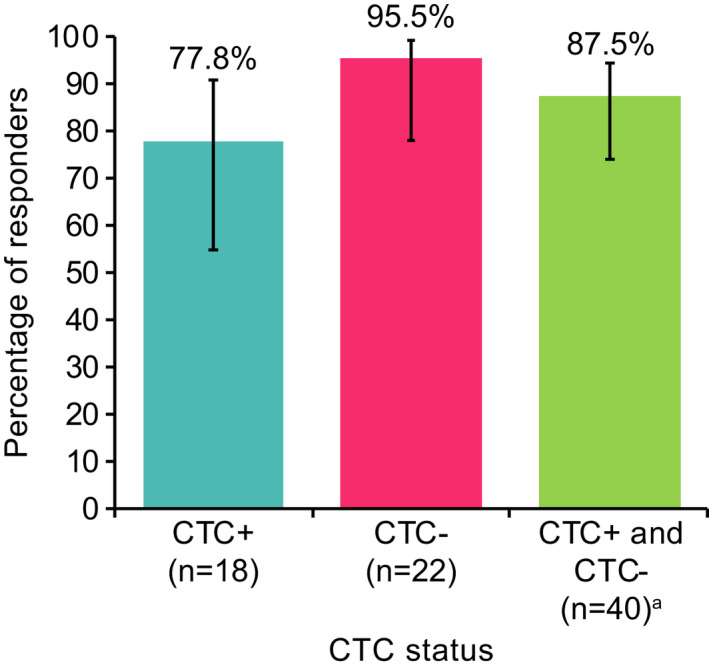
Clinical symptomatic response rates (flushing and diarrhoea) in LAN‐treated patients according to CTC status. ^a^ Two patients had missing CTC data and eight patients had missing data for clinical symptomatic response (four who were CTC+ and four who were CTC−). Error bars depict the 95% CI. CI, confidence interval; CTC, circulating tumour cell; LAN, lanreotide autogel

Circulating tumour cells data were available for 48 patients who were included in the primary endpoint analysis. Overall, 87.5% (95% CI: 73.9; 94.5) of patients achieved a clinical symptomatic response (Figure 2). Patients who were CTC− at baseline had a 5.9‐fold higher odds of achieving a symptomatic response compared with patients who were CTC+, although this was not statistically significant (odds ratio: 0.17, 95% CI: 0.02; 1.65; *p* = .126).

Overall, in addition to the study medication LAN, 18 patients were taking concomitant medications with a potential full impact on clinical symptoms, and four were taking concomitant medications with a potential partial impact on clinical symptoms (see Methods for definitions). Antipropulsives (loperamide) were taken by 18 patients, and six received subcutaneous octreotide. All patients taking concomitant medication with a potential full impact on symptoms were classed as having a clinical symptomatic response (*n* = 15 [data missing for three patients]; 5 CTC+, 10 CTC−). Of the four patients taking medication with a potential partial impact on symptoms, three were classed as having a clinical symptomatic response (2 CTC+, 1 CTC−). None of the potential influential patient characteristics (including concomitant medications potentially impacting symptoms; see Methods) were associated with clinical symptomatic response (*p* > .2 in univariate analyses).

Among those with data available, 17/22 patients (77.3%) with ENETS stage IV disease had a symptomatic response, compared with 7/7 (100.0%) with lower‐stage disease (post hoc).

### 3.3 The effect of LAN on diarrhoea and flushing

**FIGURE 3:**
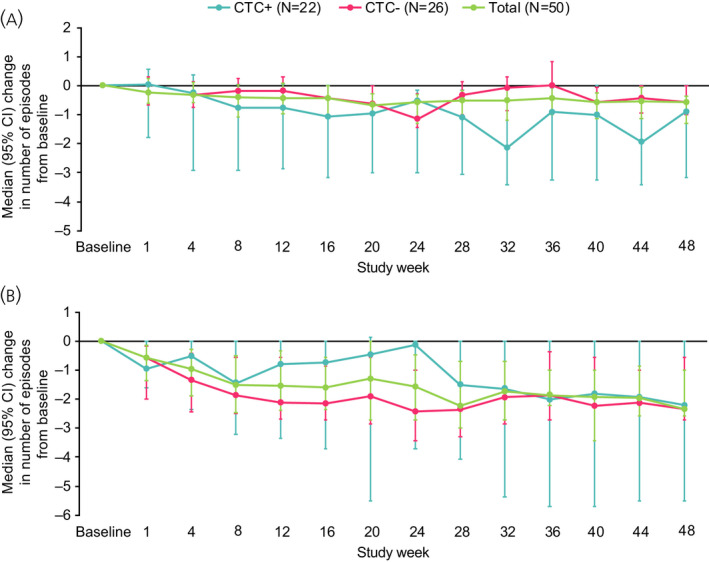
Change in number of (A) diarrhoea and (B) flushing episodes in LAN‐treated patients according to CTC status. Median data over 7 days preceding the time point are presented for all time points up to Week 12; median data from days 11–17 post‐injection are used for subsequent time points. Results based on patients with data available for symptom episodes and CTC status. Two patients had missing CTC status at baseline. Error bars depict 95% CI. CI, confidence interval; CTC, circulating tumour cell; LAN, lanreotide autogel

At baseline, the median (95% CI) daily numbers of diarrhoea and flushing episodes were 1.3 (0.6; 2.7) and 2.4 (1.5; 3.1), respectively (Table 1). Between baseline (average of 7 days before first LAN injection) and the end of the study (average of Days 11–17 after last LAN injection), median (95% CI) changes in the number of diarrhoea and flushing episodes were –0.6 (–1.3; –0.3) and –2.3 (–2.6; –1.0), respectively. Among those with data available, 70.6% (24/34) of patients had a ≥50% reduction in the number of diarrhoea episodes between baseline and the end of the study, and 77.1% (27/35) had a ≥50% reduction in the number of flushing episodes (post hoc). Changes in the number of diarrhoea and flushing episodes according to CTC status are shown in Figure 3.

### 3.4 PFS at 1 year

**FIGURE 4:**
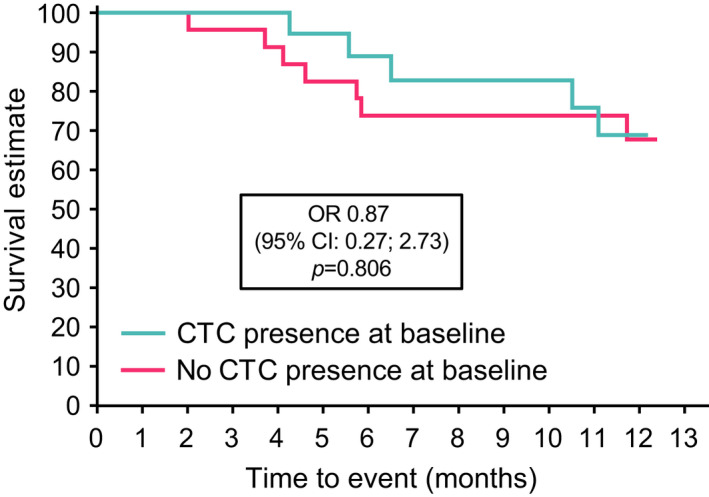
Kaplan–Meier progression‐free survival according to baseline CTC status. CI, confidence interval; CTC, circulating tumour cell; LAN, lanreotide autogel; OR, odds ratio; PFS, progression‐free survival

Among patients with CTC assessments at baseline, 12 (25.0%) had disease progression or had died at 1 year; the risk of disease progression or death was similar irrespective of CTC presence at baseline (hazard ratio of 0.87 [95% CI: 0.27; 2.73], *p* = .81) (Figure 4). The proportion (95% CI) of patients who were alive and progression free at 1 year was 66.4% (48.8; 79.2); results were similar in the CTC+ (69.0% [95% CI: 40.3; 85.9]) and CTC− (67.8 [95% CI: 43.4; 83.4) groups.

Of patients with data available (*n* = 41), the best overall response during the study was partial response in four patients (9.8%), stable disease in 29 (70.7%) and progressive disease in eight (19.5%); there were no complete responses. The proportion of partial responders was similar between CTC+ (11.1%) and CTC− (8.7%) subgroups.

### 3.5 CTC enumeration during the study

At baseline, 45.8% of patients were CTC+ (data were missing for two patients); corresponding values at weeks 4, 16, and 24, and end of study/early withdrawal were 40.0%, 27.5%, 25.6%, and 29.3%, respectively. Mean CTC levels declined up to week 24; however, the data were variable. There was no overall percentage change in CTC values; at weeks 4, 16, 24 and end of study/early withdrawal, mean (95% CI) percentage change in CTC levels from baseline was 2.5% (−22.8; 27.8), 0.6% (−28.3; 29.6), −20.5% (−35.8; −5.3) and 71.7% (−109.9; 253.3), respectively. There was no clear pattern of CTC changes according to symptomatic or radiological response at the patient level.

### 3.6 HRQoL

At the end of the study, there was no overall trend toward changes in global health status or functional HRQoL measured using EORTC QLQ‐C30. Similarly, for all symptom domains (including gastrointestinal symptoms), median change from baseline was 0 and CIs were wide. HRQoL was also assessed using the NET‐specific EORTC QLQ‐G.I.NET21 questionnaire. Median scores for gastrointestinal symptoms, social function, information function or body image were stable throughout the study, with only minor changes from baseline. At the end of the study, median (95% CI) change from baseline scores showed evidence of improvement (decreased score) for endocrine symptoms (−11.0 [−22.2; 0]) and disease‐related worries (−11.1 [−33.3; 0]), and worsening (increased score) for treatment‐related symptoms (11.0 [0; 33.3]); however, the CIs were wide.

### 3.7 Biomarker analyses

**FIGURE 5:**
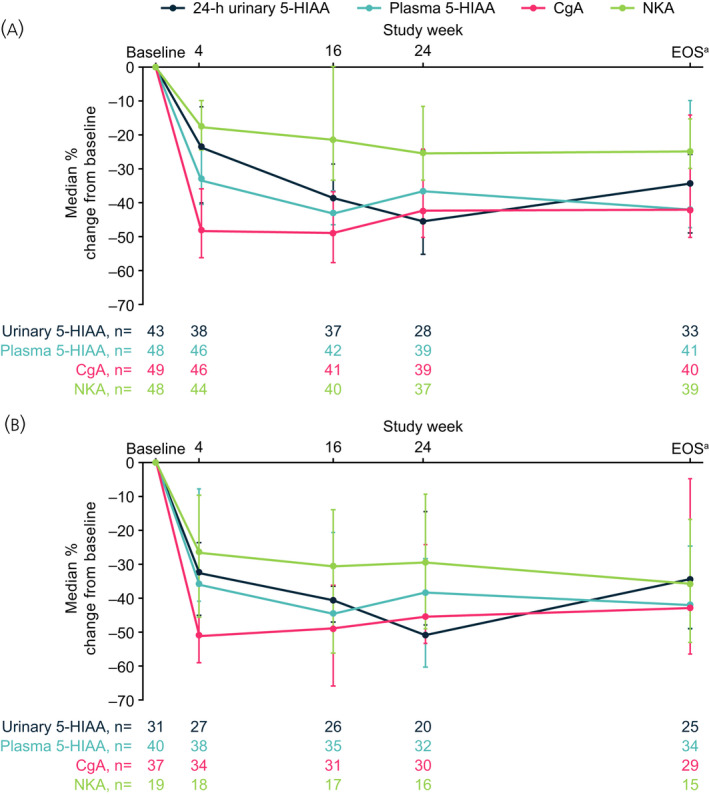
Percentage change in biomarker levels (×ULN) during treatment with LAN. (A) Overall patient population and (B) subgroup with elevated baseline levels >2 × ULN. ^a^Or early withdrawal; Note: 10 patients did not complete the study and some patients did not have biomarker data available at all visits (missing data were not imputed). Error bars depict 95% CI. Median (95% CI) percent change from baseline in CTC values was 0% (0; 0) across all time points. 5‐HIAA, 5‐hydroxyindoleacetic acid; CgA, chromogranin A; CI, confidence interval; EOS, end of study; h, hour; LAN, lanreotide autogel; NKA, neurokinin A; ULN, upper limit of normal

**FIGURE 6:**
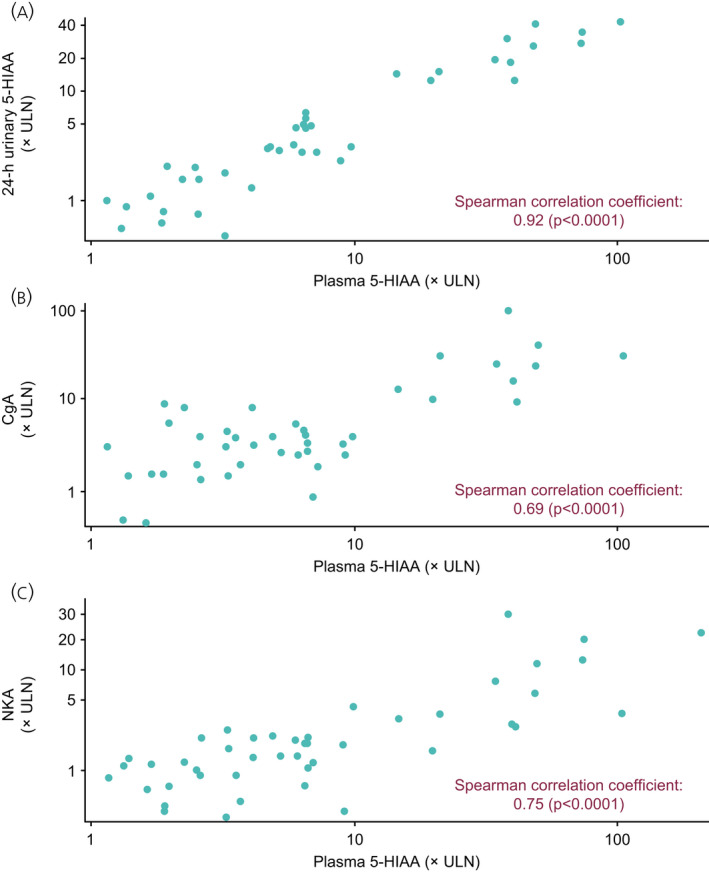
Correlation between baseline levels of plasma 5‐HIAA and (A) 24‐h urinary 5‐HIAA, (B) CgA and (C) NKA. 5‐HIAA, 5‐hydroxyindoleacetic acid; CgA, chromogranin A; NKA, neurokinin A; ULN, upper limit of normal

Baseline biomarker levels are summarized in Table 2. During LAN treatment, median values for biomarkers were reduced both in the overall population and in those with elevated levels (>2 × ULN) at baseline (Figure 5). The reduction in biomarker levels was rapid at week 4 and this reduction was sustained until end of study/early withdrawal (median percentage change from baseline at week 4 and end of study/early withdrawal, respectively, urinary 5‐HIAA [µmol/day]: −23.7% and −34.4%; plasma 5‐HIAA [ng/ml]: −33.4% and −41.9%; CgA [× ULN]: −48.3% and −42.0%; NKA [× ULN]: −17.7% and −25.0%). There were no correlations between any biomarker concentration at any visit and CTC presence at baseline (Wilcoxon test, all *p* > .2; data on file). However, post hoc analyses demonstrated that there was a strong correlation between plasma and urinary 5‐HIAA throughout the study (Spearman’s correlation coefficient: ≥0.87 at all time points [*p* < .001]; Figure 6A and Table S1). Correlations were also strong between plasma 5‐HIAA/CgA throughout the study (Spearman correlation coefficient: ≥0.78 at all time points [*p* < .0001]; Figure 6B and Table S1), but less strong for plasma 5‐HIAA/NKA (Spearman correlation coefficient: ≥0.57 at all time points [*p* < .0001]; Figure 6C and Table S1).

**TABLE 2:** Baseline biomarker levels

Biomarker	Overall population	Patients with levels >2 × ULN
Urinary 5‐HIAA (μmol/day) Median (95% CI) [*n*] × ULN [*n*]	124.0 (92.6; 222.6) [43] 3.1 (2.3; 5.6) [43]	196.9 (124.0; 603.6) [31] 4.9 (3.1; 15.1) [31]
Plasma 5‐HIAA (ng/ml) Median (95% CI) [*n*] × ULN [*n*]	436.0 (286.0; 627.0) [48] 6.2 (4.1; 9.0) [48]	462.0 (365.0; 1020.0) [40] 6.6 (5.2; 14.6) [40]
CgA (μg/L) Median (95% CI) [*n*] × ULN [*n*]	583.1 (450.8; 808.5) [49] 4.0 (3.1; 5.5) [49]	798.7 (583.1; 1455.3) [37] 5.4 (4.0; 9.9) [37]
NKA (pg/ml) Median (95% CI) [*n*] × ULN [*n*]	32.0 (24.0; 43.0) [48] 1.6 (1.2; 2.2) [48]	73.0 (54.0; 223.0) [19] 3.7 (2.7; 11.2) [19]

Abbreviations: 5‐HIAA, 5‐hydroxyindoleacetic acid; CgA, chromogranin A; CI, confidence interval; NKA, neurokinin A; ULN, upper limit of normal.

### 3.8 Safety and tolerability

Overall, most treatment‐emergent AEs (TEAEs) were mild or moderate in severity (Table 3), and only two of the TEAEs leading to discontinuation were considered treatment‐related by the investigator (bowel obstruction in one patient and a lump at the injection site in another patient). The patient who experienced a bowel obstruction died from multi‐organ failure following complications with surgery (ischemia) to treat the bowel obstruction. In addition, there were two further patients with TEAEs resulting in death during the study: one patient died of multi‐organ failure following a severe myocardial infarction and pneumonia, and another patient, with a history of ischemic heart disease and carcinoid heart disease, died as a result of heart failure. No cause of death was deemed to be treatment‐related by the investigator; however, the concomitant event of bowel obstruction in one patient was considered to be related. An additional death occurred prior to treatment administration as a result of disease progression. The most common treatment‐related TEAEs were abdominal pain, diarrhoea and injection site mass (all 10%; Table 4). Treatment‐related serious TEAEs occurred in two patients; bowel obstruction in one, and abdominal pain, nausea and vomiting in another. LAN treatment was discontinued in the patient with bowel obstruction, but continued in the latter patient, whose gastrointestinal events resolved.

**TABLE 3:** LAN TEAE profile (intensity, causality)

TEAE	Total (*n* = 50)
Any TEAE	50 (100) [509]
Intensity of TEAEs	
Mild	47 (94.0) [297]
Moderate	39 (78.0) [171]
Severe	19 (38.0) [41]
Causality of TEAEs	
Related	23 (46.0) [52]
Not related	50 (100) [456]
Missing	1 (2.0) [1]
Intensity and causality of TEAEs	
Mild and related	20 (40.0) [33]
Moderate and related	8 (16.0) [14]
Severe and related	3 (6.0) [5]
Missing and related	0
TEAEs leading to treatment discontinuation	5 (10.0) [8]
TEAEs leading to study withdrawal	4 (8.0) [4]
TEAEs leading to death	3 (6.0)^a^ [3]
Serious TEAEs	12 (24.0) [34]

Note

Data are presented as number of patients (percentage of patient) [number of events]. Patients with several AEs of different intensities or causalities are counted once for each intensity/causality concerned.

LAN, lanreotide autogel; TEAE, treatment‐emergent adverse event.

^a^ None were related to treatment.

**TABLE 4:** Treatment‐related TEAEs occurring in >1 patient treated with LAN

Treatment‐related TEAEs	Total (*n* = 50)
Gastrointestinal	
Abdominal pain	5 (10.0)
Diarrhoea	5 (10.0)
Vomiting	3 (6.0)
Steatorrhoea	2 (4.0)
Upper abdominal pain	2 (4.0)
Flatulence	2 (4.0)
Nausea	2 (4.0)
General disorders and administration site conditions	
Injection site mass	5 (10.0)
Injection site pain	2 (4.0)
Metabolism and nutrition disorders	
Decreased appetite	2 (4.0)
Nervous system disorders	
Dizziness	2 (4.0)

Abbreviations: LAN, lanreotide autogel; TEAE, treatment‐emergent adverse event.

## 4 DISCUSSION

In this phase IV, prospective, open‐label study, the majority (>85%) of patients with functional midgut NETs achieved a symptomatic response (≥50% reduction in daily diarrhoea and/or flushing episodes, and/or a decrease in the severity of flushing, by at least one level) following treatment with LAN. There was no statistical difference in symptomatic response rate between patients with and without baseline CTCs. This result may suggest that the ability of LAN to reduce secretion of serotonin and consequently alleviate the symptoms of increased serotonin secretion, such as diarrhoea and flushing, is unlikely to be related to CTC levels pretreatment. Approximately half of patients were CTC+ at baseline, which is similar to results previously reported.^13^, ^15^ A higher proportion of patients with ENETS stage IV disease had CTCs at baseline versus patients with earlier‐stage disease; no overall percentage change in CTC levels during the study was detected, however, data were variable.

In this study, two‐thirds of patients had progression free NETs at 1 year; however, there was no notable effect of CTC presence at baseline on PFS in patients receiving LAN treatment. These results differ from previous studies in NETs, where CTCs status prior to treatment was shown to be predictive of PFS and overall survival.^13^, ^15^, ^23^ However, interpretation of the PFS data is limited for three reasons, the small sample size, duration of follow‐up (1 year, which is relatively short for midgut NETs because they are often slow growing in nature) and in contrast to previous studies, the present study did not include patients with nonfunctional NETs. The robust effect of LAN on PFS in patients with nonfunctional enteropancreatic NETs has been previously demonstrated in the CLARINET study, which reported an estimated rate of PFS at 24 months of 65.1% in patients treated with LAN compared with 33.0% in those receiving placebo.^20^ In light of these findings, future studies should examine both functional and nonfunctional NETs to evaluate whether CTCs are more relevant in the nonfunctional setting.

The effect of LAN treatment on biomarkers in functional midgut NETs was also analysed in the present study with a consistent and sustained reduction in all tumour biomarker levels observed. None of the assessed biomarkers correlated with CTC presence at baseline or throughout the study period. Additionally, results presented here provide further evidence that plasma 5‐HIAA levels correlate with 24‐h urinary 5‐HIAA, as reported in previous single‐sample studies.^24^, ^25^ The collection of plasma as an alternative biological sample could allow clinical practice to move away from 24‐h urine sample collection, which can be inconvenient, cumbersome and easily compromised in cases of diarrhoea.^26^, ^27^ Plasma 5‐HIAA also correlated with CgA and NKA. The search for a novel specific biomarker for use in clinical practice is of great interest in the prediction of treatment response in NETs, as many of the existing single‐analyte markers have technical or biological complications. The use of circulating NET transcripts (NETest) has shown promising results by identifying stable and progressive disease in response to SSA treatment, at an earlier time point than using CgA.^28^, ^29^

The tolerability profile observed with LAN was similar to that observed in clinical trials, with gastrointestinal TEAEs being most frequently reported, and no new safety signals observed. Furthermore, the results presented here demonstrate that there was no deterioration in HRQoL with LAN treatment, which, in some cases, had a positive impact on HRQoL with results showing a potential improvement in endocrine symptoms. As a result of the small number of patients, interpretation of changes in HRQoL parameters was limited.

### 4.1 Strengths and limitations

Strengths of the CALM‐NET study include the prospective design, and adequately powered evaluation. The study was limited as a result of the single‐arm nature of the study design, and, therefore, lack of control group, and the small sample size available, resulting in variability. Additionally, the 40% difference in response rates between CTC+ and CTC− populations used to calculate an appropriate sample size was hypothetical in this exploratory study, and based on achieving statistical power should the relationship between those populations be clear, while taking into account practical considerations. Furthermore, there were relatively few diarrhoea episodes at baseline, which might be attributable to previous treatment with anti‐diarrhoeal medications. The assessment of diarrhoea was based on the number of episodes only; stool consistency, number, urgency and impact were not evaluated. Steatorrhoea is a known side effect of SSAs in the early period following administration which may have confounded the patient assessment of the number of diarrhoea episodes. Symptomatic assessments from week 16 onwards were conducted between 11 and 17 days after injection in order to reduce these confounding effects. The study was also limited in that assessment of the severity and number of flushing episodes is not a validated measure of symptomatic response, though it has shown utility in other published studies.^30^, ^31^ The follow‐up period was relatively short (1 year) to fully evaluate the correlation between CTC change and progression as midgut NETs are slow growing, and the study was not powered to formally assess this. Despite these limitations, CALM‐NET is one of only a few studies evaluating the prognostic significance of CTCs in patients with NETs, and the only study to date assessing disease outcome in relation to CTCs in patients with functional NETs. It is also the first study to evaluate the longitudinal change in CTCs in patients with NETs.

## 5 CONCLUSIONS

The results from the CALM‐NET study demonstrate that a clinical symptomatic response was achieved for the majority of patients with midgut NETs treated with LAN. The safety profile of LAN was consistent with previous studies, with no new safety signals observed. The symptomatic response did not statistically differ between those patients with baseline CTCs and those without. However, we acknowledge that the study was limited due to its small sample size and short follow up. In addition, a strong correlation between urinary 5‐HIAA and plasma 5‐HIAA concentrations was observed. This provides evidence of concordance between plasma 5‐HIAA and 24‐h urinary 5‐HIAA measurements in patients with NETs.

## CONFLICT OF INTEREST

T.M. has received consultancy fees from Ipsen. M.C. has received speaker and advisory board honoraria and research funding from AAA, Novartis and Ipsen. M.K. has received consultancy fees, speaker fees and travel sponsorship from Ipsen, Novartis, BMS and AAA. C.T. has participated in advisory boards for, and received educational grants from, Ipsen, AAA and Novartis. S.S. has received travel sponsorship from Novartis and consultancy fees from Faron Pharmaceuticals. J.K.R. has received speaker fees and research funds from Ipsen, Novartis and AAA. T.S. has received research funding from Ipsen and Novartis, joint work projects for Ipsen and AAA, and speaker honoraria from Novartis, and AAA. A.H. and K.H. are Ipsen employees.

## AUTHOR CONTRIBUTIONS

**Tim Meyer:** Conceptualization; Formal analysis; Investigation; Writing – original draft; Writing – review & editing. **Martyn Caplin:** Conceptualization; Formal analysis; Methodology; Writing – original draft; Writing – review & editing. **Mohid S Khan:** Conceptualization; Formal analysis; Writing – original draft; Writing – review & editing. **C. Toumpanakis:** Writing – original draft; Writing – review & editing. **Shishir Shetty:** Data curation; Investigation; Writing – original draft; Writing – review & editing. **John Ramage:** Data curation; Formal analysis; Investigation; Writing – original draft; Writing – review & editing. **Aude Houchard:** Formal analysis; Writing – original draft; Writing – review & editing. **Kate Higgs:** Conceptualization; Formal analysis; Investigation; Writing – original draft; Writing – review & editing. **Tahir Shah:** Conceptualization; Formal analysis; Investigation; Writing – original draft; Writing – review & editing.

## Supporting information

Table S1

## Data Availability

Where patient data can be anonymized, Ipsen will share all individual participant data that underlie the results reported in this article with qualified researchers who provide a valid research question. Study documents, such as the study protocol and clinical study report, are not always available. Proposals should be submitted to DataSharing@Ipsen.com and will be assessed by a scientific review board. Data are available beginning 6 months and ending 5 years after publication; after this time, only raw data may be available.
