# Supplementary material for: Circulating tumour cells and tumour biomarkers in functional midgut neuroendocrine tumours
Source: J Neuroendocrinol. 2022 Feb 7;34(4):e13096. doi: 10.1111/jne.13096 (PMC9285714; doi:10.1111/jne.13096)
Supplement: Supplementary file 1 — Table S1 [file JNE-34-0-s001.docx]

**Supplementary information for:**

**Circulating tumor cells and tumor biomarkers in functional midgut neuroendocrine tumors**

Tim Meyer,^1,2^ Martyn Caplin,^2^ Mohid S Khan,^3^ Christos Toumpanakis,^1^ Shishir Shetty,^4^ John K Ramage,^5^ Aude Houchard,^6^ Kate Higgs,^7^ Tahir Shah^4^

^1^University College London, London, UK; ^2^Royal Free Hospital, London, UK; ^3^University Hospital of Wales, Cardiff, UK; ^4^Queen Elizabeth Hospital Birmingham, Birmingham, UK; ^5^Kings College Hospital, London and Hampshire Hospitals, London, UK; ^6^Ipsen Pharma, Boulogne-Billancourt, France; ^7^Ipsen, Slough, UK

**Supplementary Table 1. Spearman correlation coefficients for plasma 5-HIAA and other biomarkers during treatment with LAN**

|  | **Baseline** | **Week 4** | **Week 16** | **Week 24** | **EOS/early withdrawal** | ***P* value** |
| --- | --- | --- | --- | --- | --- | --- |
| **Urinary  5-HIAA [n]** | 0.924 [40] | 0.906 [42] | 0.871 [40] | 0.902 [31] | 0.886 [38] | < 0.0001 at all time points |
| **CgA [n]** | 0.690 [46] | 0.872 [46] | 0.783 [41] | 0.783 [40] | 0.861 [41] | < 0.0001 at all time points |
| **NKA [n]** | 0754 [45] | 0.701 [44] | 0.572 [41] | 0.604 [39] | 0.639 [41] | < 0.0001 at all time points |

5-HIAA, 5-hydroxyindoleacetic acid; CgA, chromogranin A; EOS, end of study; LAN, lanreotide autogel; NKA, neurokinin
